# Cyclophilin D Knock-Out Mice Show Enhanced Resistance to Osteoporosis and to Metabolic Changes Observed in Aging Bone

**DOI:** 10.1371/journal.pone.0155709

**Published:** 2016-05-16

**Authors:** Laura C. Shum, Noelle S White, Sergiy M. Nadtochiy, Karen L. de Mesy Bentley, Paul S Brookes, Jennifer H. Jonason, Roman A. Eliseev

**Affiliations:** 1 Center for Musculoskeletal Research, University of Rochester School of Medicine and Dentistry, Rochester, New York, United States of America; 2 Department of Anesthesiology, University of Rochester School of Medicine and Dentistry, Rochester, New York, United States of America; 3 Department of Pathology and Laboratory Medicine, University of Rochester School of Medicine and Dentistry, Rochester, New York, United States of America; Louisiana State University, UNITED STATES

## Abstract

Pathogenic factors associated with aging, such as oxidative stress and hormone depletion converge on mitochondria and impair their function via opening of the mitochondrial permeability transition pore (MPTP). The MPTP is a large non-selective pore regulated by cyclophilin D (CypD) that disrupts mitochondrial membrane integrity. MPTP involvement has been firmly established in degenerative processes in heart, brain, and muscle. Bone has high energy demands and is therefore expected to be highly sensitive to mitochondrial dysfunction. Despite this, the role of mitochondria and the MPTP in bone maintenance and bone pathology has not been elucidated. Our goal was to determine whether mitochondria are impaired in aging bone and to see if protecting mitochondria from MPTP opening via CypD deletion protects against bone loss. We found that bone mass, strength, and formation progressively decline over the course of 18 months in C57BL/6J mice. Using metabolomics and electron microscopy, we determined that oxidative metabolism is impaired in aging bone leading to a glycolytic shift, imbalance in nucleotides, and decreased NAD^+^/NADH ratio. Mitochondria in osteocytes appear swollen which is a major marker of MPTP opening. CypD deletion by CypD knockout mouse model (CypD KO) protects against bone loss in 13- and 18-month-old mice and prevents decline in bone formation and mitochondrial changes observed in wild type C57BL/6J mice. Together, these data demonstrate that mitochondria are impaired in aging bone and that CypD deletion protects against this impairment to prevent bone loss. This implicates CypD-regulated MPTP and mitochondrial dysfunction in the impairment of bone cells and in aging-related bone loss. Our findings suggest mitochondrial metabolism as a new target for bone therapeutics and inhibition of CypD as a novel strategy against bone loss.

## Introduction

Aging is a complex phenomenon marked by deterioration in form and function of many tissues. It is a multifactorial process with well-established hallmarks, such as loss of hormonal stimulation and increased oxidative stress. These and other age-associated factors converge on mitochondria, and disrupt their function [1]. As mitochondria deteriorate, they lose capacity for oxidative phosphorylation (OxPhos), biosynthesis, and ion transport. The relationship between mitochondrial dysfunction and aging has been studied in many tissues, including the brain, muscle, and cardiovascular system [2–7], but not bone. Since bone tissue has high energy demands due to continuous remodeling, intact and functioning mitochondria are important to bone health.

The most common cause of mitochondrial dysfunction is increased activity of the mitochondrial permeability transition pore (MPTP), a large, non-selective mitochondrial pore that opens in response to stresses such as excessive calcium, reactive oxygen species, and hormone depletion [8–10]. MPTP opening dissipates the mitochondrial membrane potential, inducing mitochondrial swelling [11]. MPTP-mediated mitochondrial dysfunction has been associated with cell death during aging and other pathologies in many tissues [11–13]. The MPTP is opened by its protein regulator, cyclophilin D (CypD), a chaperone that also assists in protein folding. Deletion of CypD protects mitochondrial function and prevents necrotic cell death, by preventing opening of the MPTP [14]. In fact, CypD deletion via global knockout (KO) in mice showed beneficial results in models of Alzheimer's disease and cardiac reperfusion injuries [15,16]. Despite this, CypD deletion has not been investigated in bone pathologies.

Reduced bone mass and mineral density are common characteristics in aging bone, leading to a significant increase of fracture risk in the elderly. In addition to hormone depletion, oxidative stress has come into focus as a second major pathogenic factor in age-associated bone loss [17,18]. Mitochondria are both a major source and major sensor of oxidative stress and are sensitive to changes in hormonal stimuli; however their role in aging bone has not been thoroughly studied.

Our recent work and studies from other groups highlight the importance of mitochondrial oxidative phosphorylation in osteogenic cells [19–21]. Therefore, osteogenically differentiating bone marrow stem/stromal cells (BMSCs) and their progeny, osteoblasts (OB) and osteocytes (OT), are expected to be especially sensitive to mitochondrial dysfunction, leading to decreased bone formation and increased bone loss. Considering the multifactorial nature of aging and the fact that aging-related stresses converge on mitochondria via the CypD-mediated MPTP, we tested the hypothesis that mitochondrial dysfunction and the MPTP are involved in bone loss. Using metabolomics, electron microscopy and a global CypD knockout mouse model (CypD KO), we found that CypD deletion protects mitochondrial function and is beneficial for aging bone.

## Materials and Methods

### Materials

Chemicals were from Sigma unless otherwise noted. Cell culture media was from Invitrogen or Stem Cell Technologies.

### Animals

C57BL/6J wild type mice were from the National Institute of Aging and were acclimated at our vivarium facility. CypD KO mice (C57BL/6J background) were a kind gift from Dr. George Porter (University of Rochester) [22]. CypD KO mice were backcrossed to C57BL/6J for five generations. CypD KO mice were not significantly different in size compared to the C57BL/6J mice. Male mice were used for experiments. Animal husbandry and experiments were performed in accordance with the Division of Laboratory Animal Medicine, University of Rochester, state and federal law, and National Institutes of Health policy. University of Rochester Institutional Animal Care and Use Committee (IACUC) specifically approved this study. Conditions of animal husbandry, i.e. food type, light schedule, cage density, etc., at NIA were similar to the conditions at our animal facility.

### Bone microCT and biomechanical testing

After sacrifice, femurs, tibiae, and spine were isolated and cleaned of soft tissue. Right femurs were wrapped in phosphate buffered saline soaked gauze and stored at -80°C to prevent drying until biomechanical testing was performed. For micro computed tomography (microCT), bones were fixed in 10% neutral buffered formalin (NBF) for 72 hours and imaged using a VivaCT 40 tomograph (Scanco Medical). A calibrating phantom was used to standardize radiodensities among scans. Volume quantification was performed using Scanco analysis software. Bone vs total volume (BV/TV), trabecular number (Tb. Number), trabecular thickness (Tb. Thickness), trabecular separation (Tb. Separation) and cortical thickness (Cort. Thickness) were determined for tibiae, femurs, and the third lumbar vertebrae of the spine (L3). For biomechanical testing, right femurs were subjected to torsion testing. Briefly, samples were held in bone cement (DePuy Orthopaedics) in aluminum holders and tested using an EnduraTec TestBench system (Bose) at a displacement rate of 1°/s until failure. The torque data were plotted against rotational deformation to determine the maximum torque, torsional rigidity, and energy to maximum.

### Histology

NBF-fixed samples were processed for histology via decalcification in EDTA for two weeks followed by paraffin embedding. Sections were cut to 5 μm and stained with Alcian Blue/Hematoxylin and Orange G (ABH/OG).

### Histomorphometry

ABH/OG stained slides were visualized using an Axioscope 40 (Zeiss) microscope equipped with an Olympus DP72 camera (Olympus) and evaluated with Osteomeasure software (OsteoMetrics). Slides were analyzed to measure trabecular bone area to total area in proximal tibia, distal femur, and L3. Three different slides were counted per mouse and averaged. There were at least five mice per age group.

### Bone formation rate

Mice were labeled via intraperitoneal injection with Alizarin Red (Day 0), followed by calcein (Day 7). Seven days after calcein injection (Day 14), mice were sacrificed. Long bones were collected, stripped of soft tissue, and either fixed in 70% ethanol and shipped to the Orthopaedic Histology and Histomorphometry Laboratory at the Yale School of Medicine for further processing, embedding into methylmethacrylate, and sectioning, or processed for frozen sectioning as this technique became available in the Center for Musculoskeletal Research at the University of Rochester. Sections were visualized using a fluorescence Axioscope 40 microscope (Zeiss) equipped with an Olympus DP72 camera (Olympus) and analyzed using ImageJ software to calculate bone formation rate (BFR) as follows: BFR = MAR x (MS/BS) where MAR = Ir.L.Th/Ir.L.t. and MS = (dLS+sLS/2)/BS (MAR, mineral apposition rate; BS, bone surface; dLS, double labelled surface; sLS, single labelled surface; Ir.L.Th, distance between labels; and Ir.L.t, time between labels). Five mice per group were analyzed.

### Osteoclast activity

Bone sections were stained for tartrate-resistant acid phosphatase (TRAP), counter-stained with FastGreen and scanned in an Olympus VS120 whole slide imager. TRAP+ OCs were assessed using Visiopharm software that calculated osteoclast surface per bone surface (Oc.S/B.S.). In addition, blood was collected and serum prepared and stored at -80°C until assayed. The bone resorption marker, CTX-I, was assessed in serum samples using the Ratlaps CTX-I EIA kit (Immune Diagnostic Systems) according to the manufacturer’s instructions. Absorbance was measured at 450 nm and 650 nm. Data were analyzed using GraphPad Prism 5 (GraphPad Software, Inc) to generate a standard curve and determine concentrations in pg/mL. Five mice per group were analyzed.

### Metabolomics

Samples for metabolomic experiments were prepared as follows: bone tissue was cleaned of soft tissue, bone marrow, cartilage, and periosteum, then flash frozen in liquid nitrogen. Equal quantities of tissue were pulverized in liquid nitrogen and metabolites were extracted in 5 ml of 80% methanol. Extracts were dried under nitrogen stream and reconstituted in 200 μl of 50% methanol, and then analyzed using reverse phase liquid chromatography (LC) with an ion-pairing reagent in a Shimadzu HPLC coupled to a Thermo Quantum triple-quad mass spectrometer (MS). Data were analyzed using Mzrock machine learning tool kit (http://code.google.com/p/mzrock/), which automates analysis of targeted metabolomics data based on chromatographic retention time, whole molecule mass, collision energy, and resulting fragment mass. Data are presented as fold change over 3-mo-old mice. Three mice per group were analyzed.

### Electron microscopy

Mice were perfused with 2.5% glutaraldehyde in 0.1 M sodium cacodylate buffer and bones were dissected, fixed for 24 hours, and decalcified as described above. Samples were post-fixed in 1.0% OsO4, dehydrated in a graded series of ethanol to 100%, and transitioned into propylene oxide for infiltration and embedding into EPON/Araldite epoxy resin. Thin sections (70nm) cut using a diamond knife were placed onto carbon/formvar slot grids, stained with aqueous uranyl acetate and lead citrate, examined and photographed using a Hitachi H-7650 transmission electron microscope with an attached Gatan Erlangshen 11 megapixel digital camera. The number of mitochondria in normal orthodox conformation and in swollen conformation was blindly counted by three investigators. An orthodox conformation is characterized by an electron-dense and crista-rich matrix, and a swollen conformation is characterized by an increased surface area and a less electron-dense matrix with fewer cristae. A total of 15 cells/sample were analyzed. Three mice per group were analyzed.

### Statistical analysis

Data were analyzed using Prism 5.01 (GraphPad Software). Mean values and standard deviation were calculated, and the statistical significance (*p* < 0.05) established using ANOVA for >2 groups of variables or Student *t*-test for 2 groups of variables.

## Results

### Decline in bone mass and strength with age in male C57BL/6J mice

**Fig 1:**
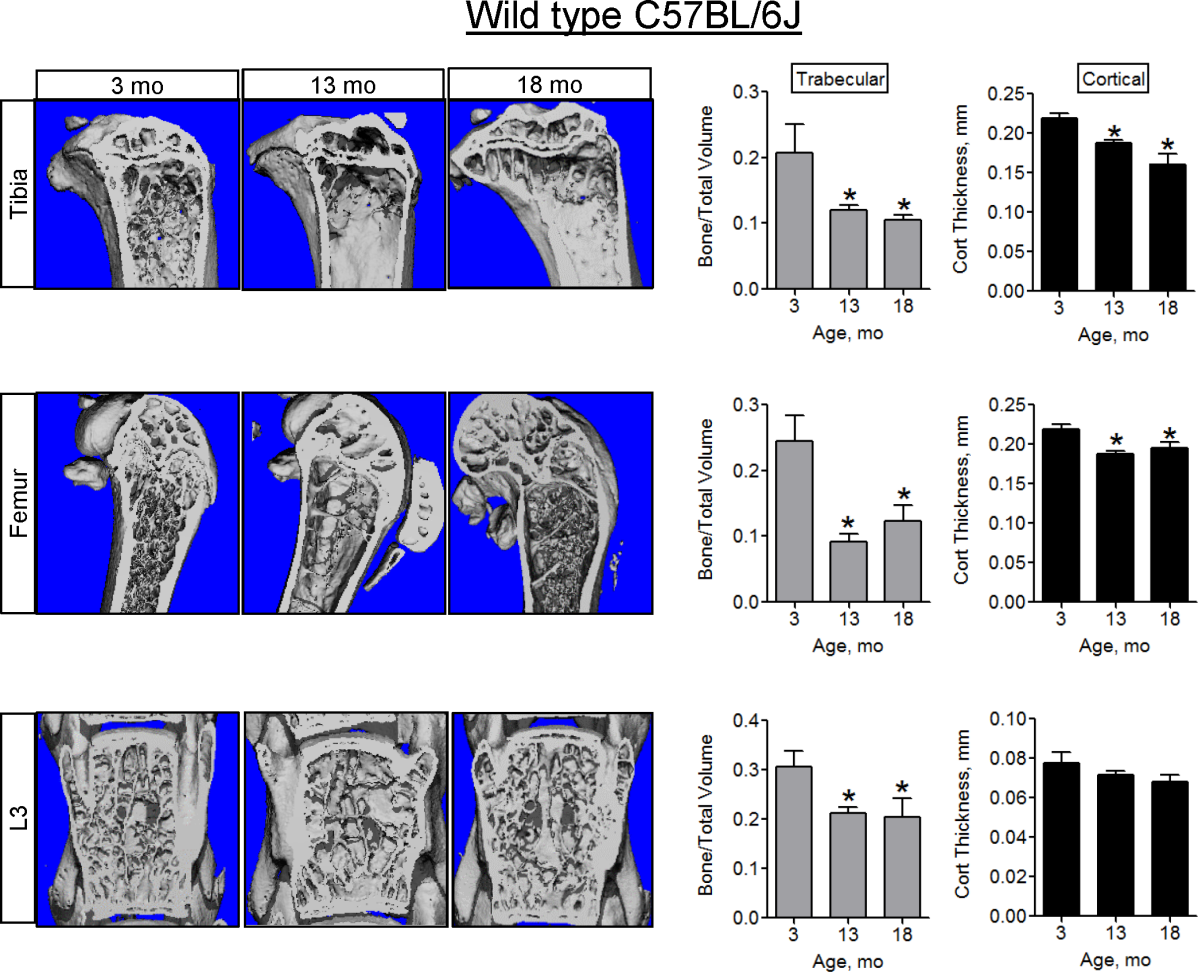
Bone loss in 13- and 18-mo-old C57BL/6J mice. Representative microCT images of mouse tibiae, femurs, and L3 from 3-, 13-, and 18-mo-old C57BL/6J mice. Graphs show quantitative volumetric analysis of microCT data. Data are Means ± SD (n = 5–15). *, *p*<0.05 vs 3 mo as determined with ANOVA.

**Fig 2:**
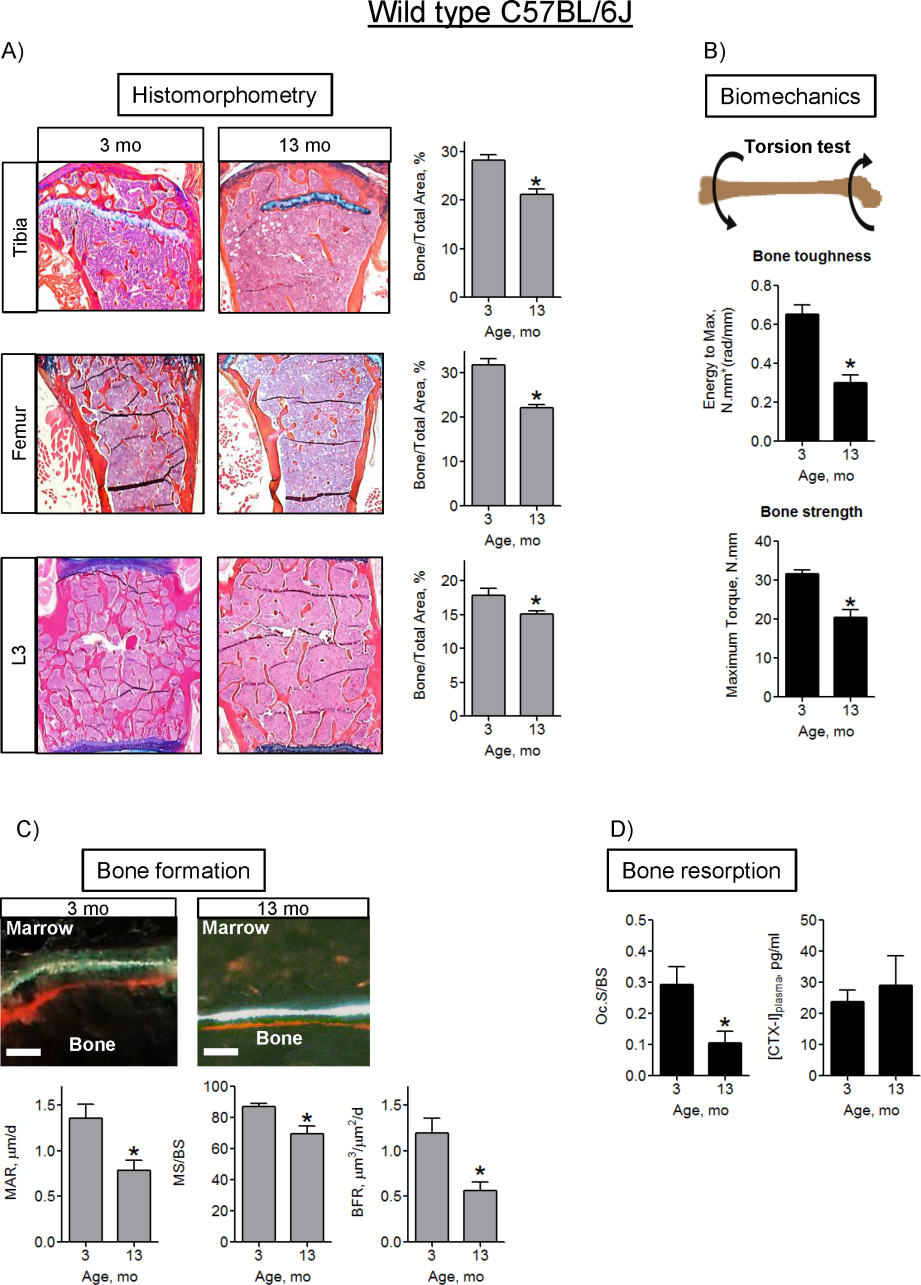
Decline in bone architecture, strength, and formation in 13-mo-old C57BL/6J mice. A) Histological evaluation of mouse bones. Sections stained with ABH/OG were analyzed using histomorphometry and OsteoMeasure software and trabecular Bone to Total Area ratios were calculated; B) Biomechanical torsion test of mouse femurs; C) Bone formation assay. Shown are representative sections and quantitative analyses of mouse bones labeled with Alizarin Red and Calcein for dynamic Mineral Apposition Rate (MAR), Mineralizing Surface/Bone Surface (MS/BS) and Bone Formation Rate (BFR) assay. Scale bar is 5 μm; D) TRAP-positive osteoclast surface relative to bone surface (Oc.S/B.S.) and CTX-I bone resorption/OC activity marker measured using ELISA. Data are Means ± SD (n = 5). *, *p*<0.05 vs 3 mo as determined with *t-*test.

In accordance with previous findings, we sought to verify bone loss seen with increased age in C57BL/6J male mice. We examined bone phenotype at ages 3-, 13-, and 18-mo by microCT (Fig 1 and S1 Fig). Data collected from the proximal tibia showed a progressive loss of trabecular bone, in addition to significant cortical thinning at both 13- and 18-mo, compared to 3-mo. Both trabecular and cortical bone from femur declined at 13- and 18-mo. In the spine (L3), only trabecular bone decreased, while cortical thickness did not significantly diminish. Since we observed significant bone loss as early as at 13-mo, which is consistent with the literature [23], we primarily focused on 13-mo old mice even though 13-mo is not considered advanced age [24]. The microCT findings were confirmed with histomorphometry. Trabecular bone to total area decreased significantly at 13-mo in the tibia, femur, and spine (Fig 2A). Subsequently, we performed biomechanical testing to determine if the functional properties of the femur were also reduced with age (Fig 2B). Both bone toughness and bone strength decreased significantly at 13-mo.

Bone homeostasis is maintained by balanced activity between bone forming osteoblasts (OB) and bone resorbing osteoclasts (OC). In aging, this balance is shifted. We quantified OB function in long bones using in vivo fluorescent double labeling. All of the measured and calculated parameters, i.e. Mineral Apposition Rate (MAR), Mineralizing Surface to Bone Surface ratio (MS/BS), and Bone Formation Rate (BFR), decreased significantly by 13-mo in trabecular bone (Fig 2C). Similar results were observed in cortical bone (S2 Fig). There were no significant changes in osteoblast numbers, proliferation, apoptosis or differentiation at 13-mo (S1 Table). Resorption was measured by histological detection of TRAP-positive osteoclast surface relative to bone surface (Oc.S/B.S.) and serum levels of bone resorption marker CTX-I. While Oc.S/B.S. declined with age, overall bone resorption did not change (Fig 2D), indicating that bone loss at this age is due to a decline in OB function, as opposed to an increase in resorption by OCs. Collectively, Figs 1 and 2 demonstrate that in agreement with previous studies on bone aging [23], we observed a significant bone loss and decline in bone formation in male C57BL/6J mice at 13- and 18-mo of age.

### Effect of age on bone metabolism and mitochondria in C57BL/6J mice

To find out if there are changes in energy metabolism in aging bone, we performed metabolomic studies using metabolite extraction and detection with Liquid Chromatography-Mass Spectroscopy (LC-MS), similar to our collaborators’ previous studies [25].

**Fig 3:**
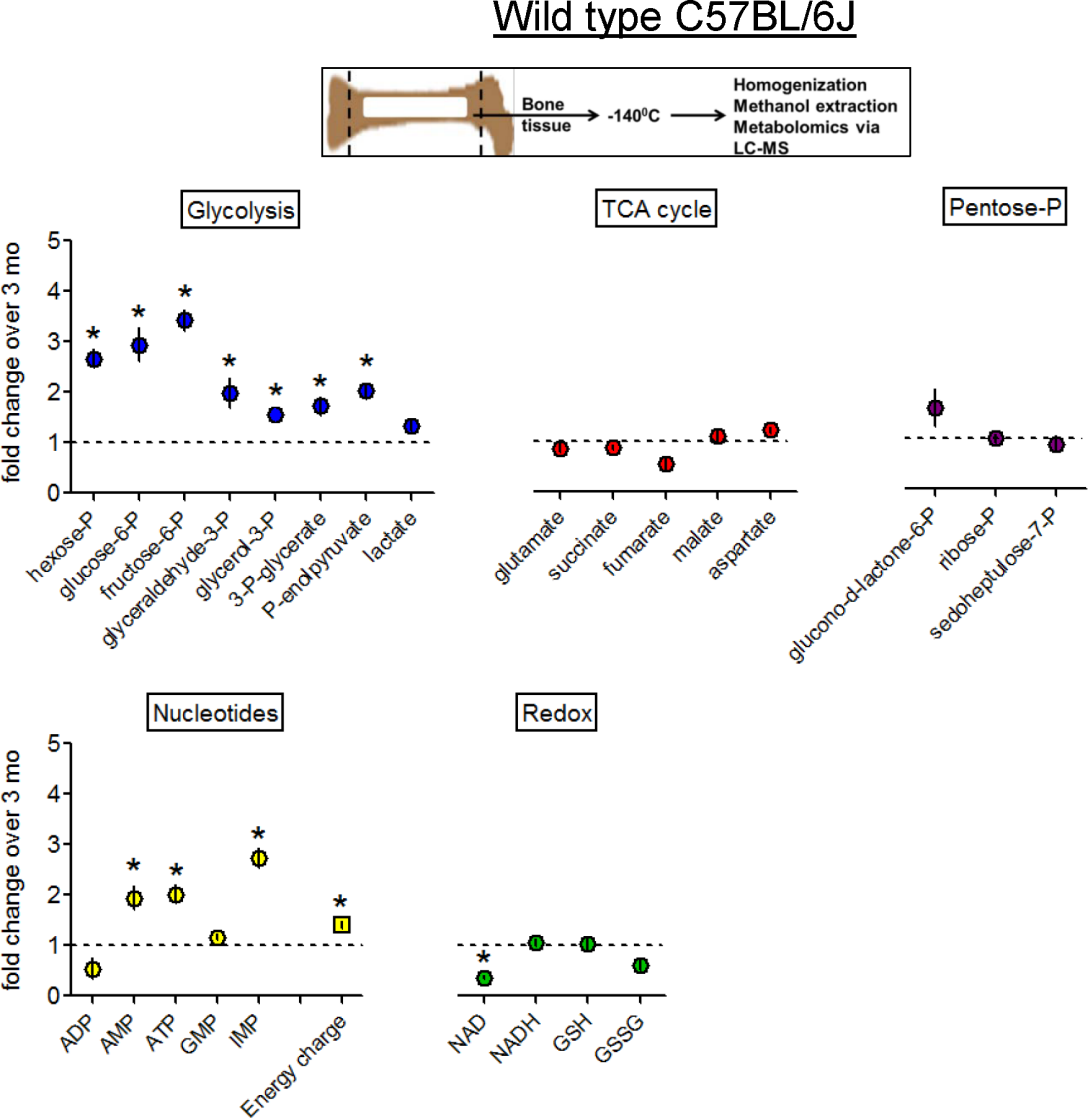
Metabolic changes in bone samples from 13-mo-old C57BL/6J mice. Small metabolites were extracted from bone shafts of tibia and femurs and analyzed using metabolomic LC-MS. Metabolites are grouped into appropriate metabolic pathways. Data are Means ± SD (n = 3). *, *p*<0.05 vs 3 mo as determined with *t*-test.

Bones of 13-mo-old mice showed a significant increase in the pool of both 6- and 3-carbon glycolytic intermediates compared to 3-mo-old mice, while mitochondrial TCA cycle intermediates were not significantly changed (Fig 3, top left and middle panels). This accumulation of glycolytic intermediates without a corresponding increase in TCA cycle intermediates likely indicates decreased mitochondrial function, as the glycolytic products are not being further utilized in mitochondria. However, glycolytic intermediates did not appear to be diverted to the non-mitochondrial Pentose Phosphate Pathway (PPP), because PPP intermediates were not significantly changed (Fig 3, top right panel).

In addition to glycolysis, PPP, and the TCA cycle, we were able to detect cellular nucleotides and redox compounds. Importantly, we detected significant elevations of both ATP and AMP in bones of 13-mo-old mice compared to 3-mo-old mice, leading to a significant increase in the Energy Charge (ATP + ½ADP/ATP + ADP + AMP) parameter (Fig 3, bottom left panel). The elevated Energy Charge is an indicator of lower cell energy demand and compromised energy metabolism [25]. NAD+/NADH ratio is known to be significantly decreased in aging [26,27], and in agreement with this, our 13-mo-old bone samples had significantly lower levels of NAD+ with similar levels of NADH (Fig 3, bottom right panel), and therefore a lower NAD+/NADH ratio compared to bone samples from 3-mo-old mice (0.639 +/- 0.111 vs 4.243 +/- 1.443, *p* = 0.011). Similar metabolic changes were observed at 18-mo with more pronounced elevation of the Energy Charge and decrease in NAD+/NADH ratio (S3 Fig) indicative of progressive decline in bone oxidative metabolism in aging. It should be noted that, similar to other studies using this metabolomic technique, we were not able to resolve all cellular metabolites; however, we were able to detect a broad representation. In sum, these data demonstrate the presence of a glycolytic shift and compromised cell energy metabolism in the bone tissue of C57BL/6J mice at 13-mo.

**Fig 4:**
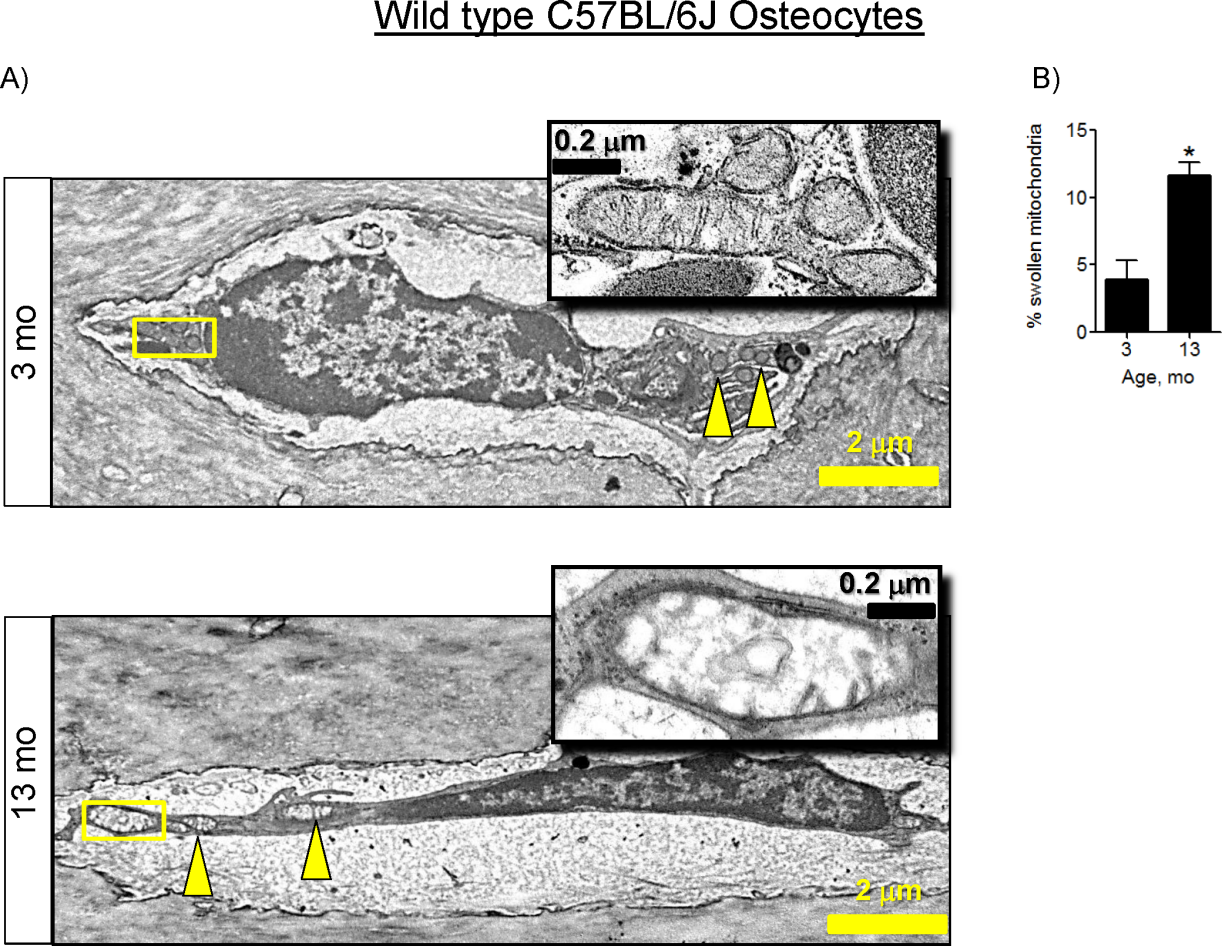
Mitochondrial swelling in osteocytes in bones from 13-mo-old C57BL/6J mice. A) Electron micrographs of osteocytes (OTs) in mouse tibia. Arrowheads indicate mitochondria. Boxed mitochondria are enlarged and shown in the insets; B) Quantitative analysis of mitochondrial morphology. Fifteen cells per sample and 3 mice per group were blindly analyzed by three independent scorers and the number of mitochondria in a swollen conformation was determined. Data are Means ± SD. *, *p*<0.05 as determined with *t*-test.

As noted above, the metabolic profile changes in bone samples from 13-mo-old mice are consistent with the changes observed after mitochondrial dysfunction in other tissues [25]. Mitochondrial function and mitochondrial morphology are tightly linked; changes in mitochondrial activity are often accompanied by changes in mitochondrial morphology and ultrastructure [28]. Therefore, we examined mitochondrial morphology in tibial bone samples using electron microscopy. We did not detect any significant changes in mitochondrial morphology in OBs (S4 Fig); however, we detected mitochondrial swelling (a marker of increased MPTP activity) in osteocytes (OTs) from 13-mo-old mice (Fig 4). Together, these data suggest that the observed loss of bone and decline in bone formation are associated with changes in bone tissue energy metabolism and mitochondrial morphology, consistent with MPTP-mediated mitochondrial dysfunction in OTs.

### CypD deletion protects against age-associated bone loss

The most common phenomenon associated with mitochondrial swelling and dysfunction is opening of the MPTP by its protein regulator CypD, however, the role of the MPTP and CypD in bone loss has not been previously described. We further investigated the role of mitochondrial metabolism in bone loss using a global CypD KO mouse model.

**Fig 5:**
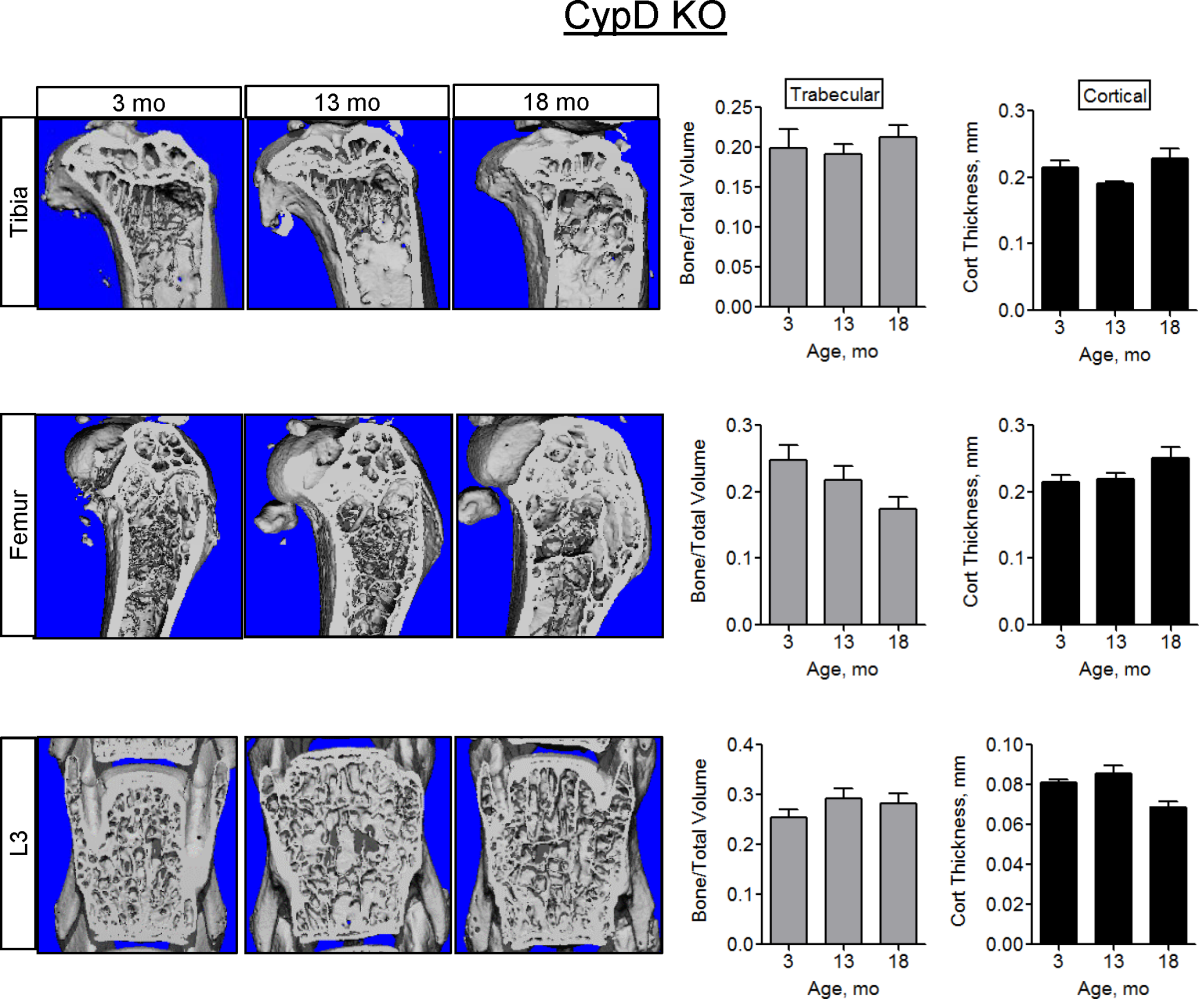
CypD KO mice do not show bone loss at 13 and 18 months of age. Representative microCT images of mouse tibiae, femurs, and L3 from 3-, 13-, and 18- mo-old CypD KO mice and graphs showing quantitative volumetric analysis of microCT data. Data are Means ± SD (n = 5–15). *, *p*<0.05 vs 3 mo as determined with ANOVA.

**Fig 6:**
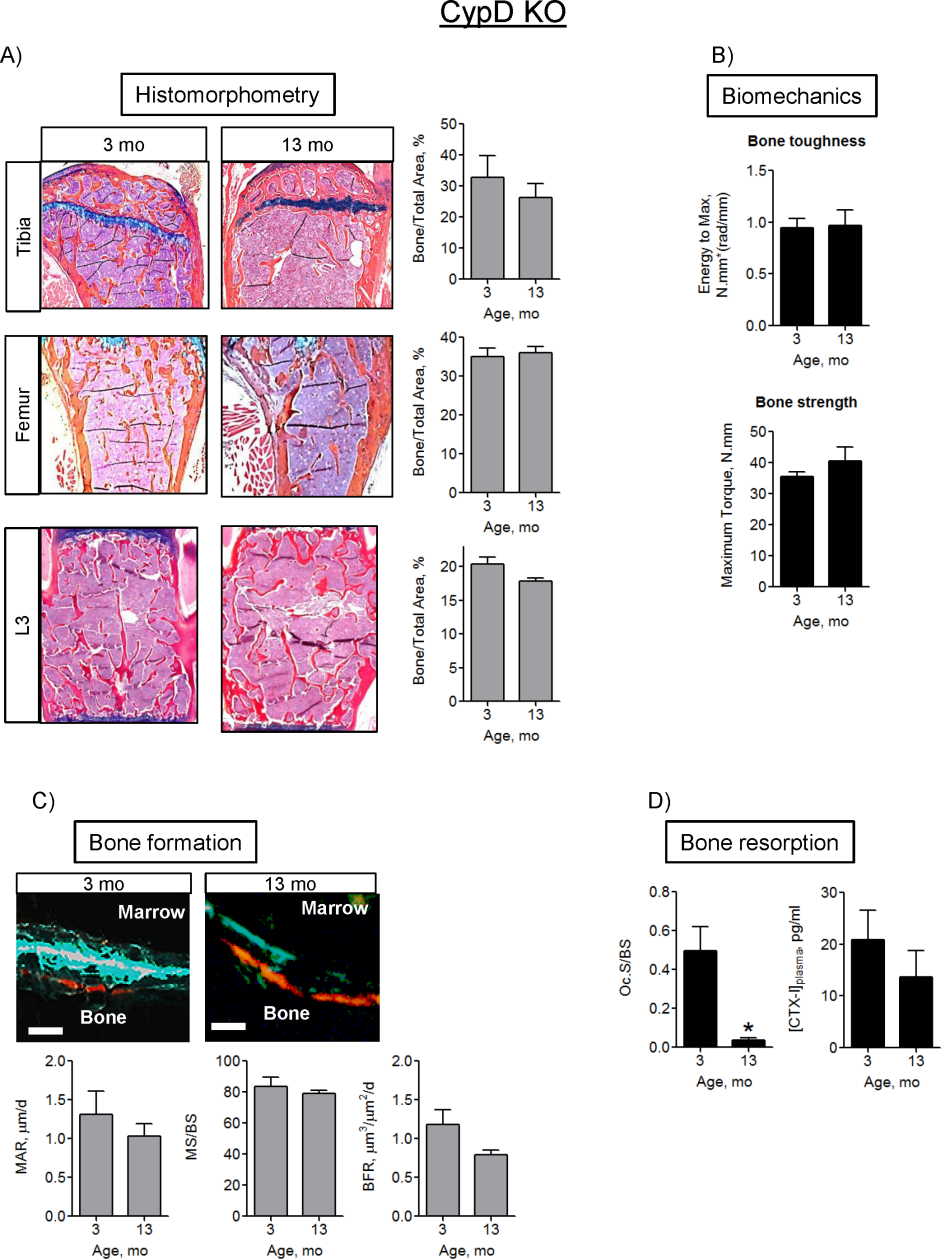
CypD deletion protects against the decline in bone architecture, strength, and formation in 13-mo-old mice. A) Histological evaluation of mouse bones, Trabecular Bone to Total Area ratios; B) Biomechanical torsion test of mouse femurs; C) Bone formation assay. Quantitative analysis of Mineral Apposition Rate (MAR), Mineralizing Surface/Bone Surface (MS/BS) and Bone Formation Rate (BFR). Scale bar is 5 μm; D) Bone resorption assay. TRAP-positive osteoclast surface relative to bone surface (Oc.S/B.S.) and CTX-I bone resorption/OC activity marker measured using ELISA. Data are Means ± SD (n = 5). *, *p*<0.05 vs 3 mo as determined with *t-*test.

Here, we assessed the bone phenotype in CypD KO mice. In contrast to the wild type C57BL/6J mice shown in Fig 1, the CypD KO mice (C57BL/6J background), show no decrease in trabecular bone volume or cortical thickness at 13- or 18-mo as measured by microCT (Fig 5). Although trabecular number decreased in the tibia and femur, there was no trabecular thinning or increased trabecular separation (S5 Fig). Since the two strains of studied mice were not littermates, we avoided direct comparison of these two groups and analyzed them for age-related changes separately. Nevertheless, it should be noted that at 3-mo of age, there were no significant differences between wild type C57BL/6J and CypD KO bones as evident from the microCT data shown in Figs 1 and 5. The observed bone-protective effect in CypD KO mice was further confirmed with histomorphometry (Fig 6A), showing no loss of trabecular Bone to Total Area ratio. Additionally, functional properties of the femur measured with biomechanical testing did not decline by 13-mo when compared to 3-mo (Fig 6B). There was no significant decline in the indices of bone formation, MAR, MS/BS, and BFR in trabecular (Fig 6C) or cortical (S6 Fig) surfaces. Bone resorption activity measured by TRAP staining does not appear to be affected by ablation of CypD at 3-mo (compare Fig 6D to Fig 2D), however there is a more pronounced decline in TRAP positive OCs in CypD KO mice at 13-mo. While this effect requires further investigation, it is not unexpected as previous studies by Miyazaki et al [29] showed inverse correlation between mitochondrial activity and OC function. Thus, improved mitochondrial function due to CypD deletion could be a reason for accelerated OC decline in aging in CypD KO mice. Similar to the wild type C57BL/6J mice, there is no effect on serum CTX-I levels with aging. Together, these data indicate that MPTP loss-of-function by CypD deletion protects against bone decline and is beneficial for bone forming function with aging.

### CypD deletion protects bone mitochondria

**Fig 7:**
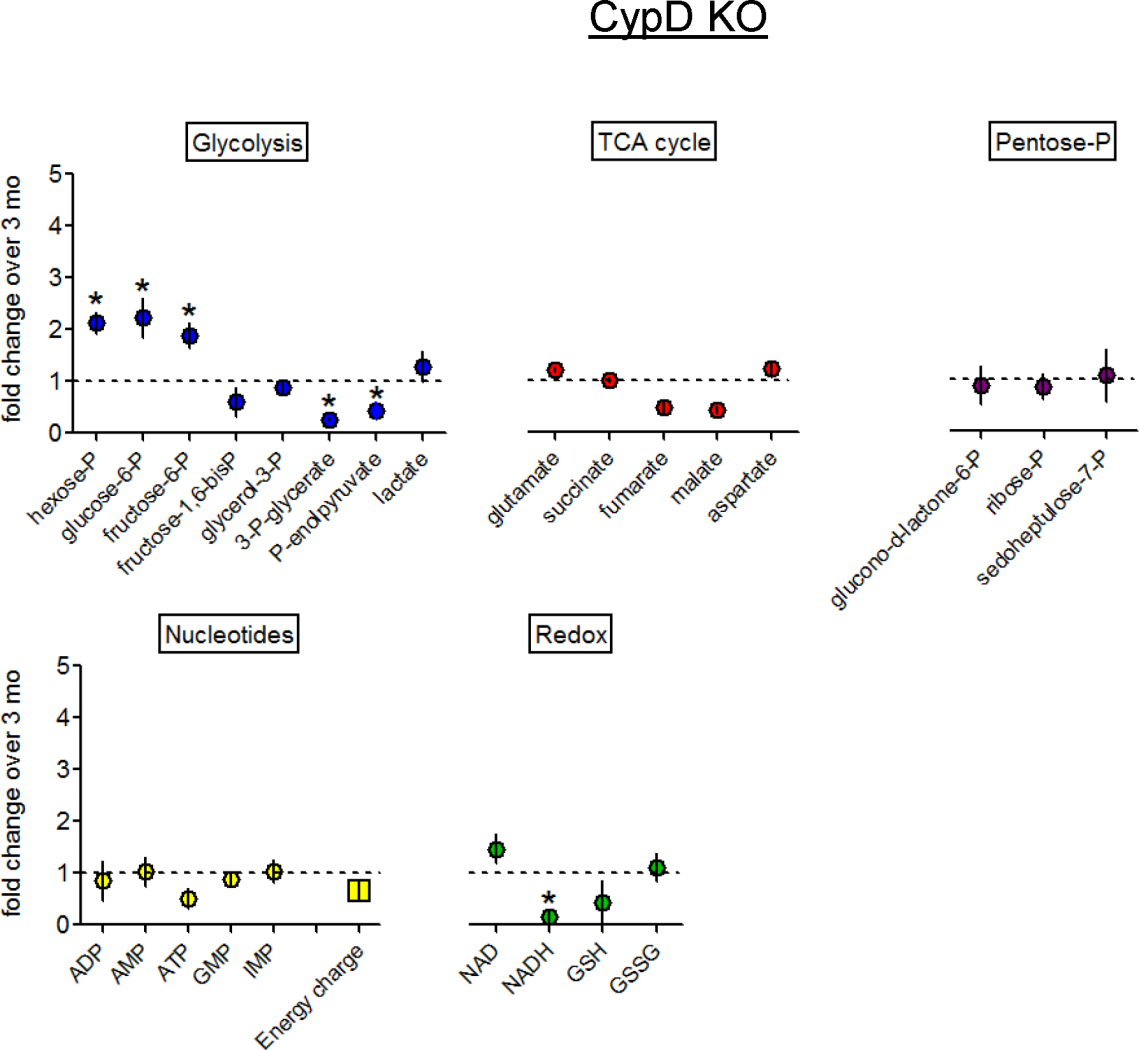
CypD deletion protects against metabolic changes in bone samples from 13-mo-old mice. Metabolomic analysis of mouse bones using MS-LC. Data are Means ± SD (n = 3). *, *p*<0.05 as determined with *t-*test.

Since an aging bone phenotype was not detected in CypD KO mice at 13 or 18 mo, we wanted to determine if energy metabolism is protected in aged CypD KO bone cells as well. We performed the same analyses as in the wild type C57BL/6J mice, i.e. metabolomics and electron microscopy. As discussed above, we avoided a direct comparison between the wild type C57BL/6J and CypD KO mice, but to make sure that there are no fundamental differences in their metabolism, we compared levels of metabolites in these mice at 3-mo of age and found no significant differences (S2 Table). We then evaluated the effect of aging on the bone cell metabolome in 13-mo-old CypD KO mice. Fig 7 (top left panel) demonstrates that in contrast to the wild type C57BL/6J mice, there was no glycolytic shift in bone samples from 13-mo-old CypD KO mice. Although some 6-carbon glycolytic intermediates were elevated, most 3-carbon intermediates were significantly lower when compared to 3-mo-old CypD KO mice. There were no significant changes in the TCA cycle intermediates, nucleotides, or the energy charge (Fig 7, top middle and right panels) indicating that there were no obvious metabolic disturbances in these tissue samples when compared to 3-mo-old. There was a decrease in NADH levels, likely indicating that the mitochondrial electron transport chain and/or NADH consuming enzymes are active and mitochondria are healthy and functional (Fig 7, bottom left panel). The NAD+/NADH ratio was not significantly changed in 13- versus 3-mo bone (6.548 +/- 1.714 vs 4.124 +/- 2.984, *p* = 0.520) again indicating a general lack of metabolic disturbances associated with aging [26,27]. The PPP intermediates were unchanged (Fig 7, bottom right panel). At 18-mo, CypD KO mouse bone metabolome was still not significantly different from that at 3-mo (S7 Fig).

**Fig 8:**
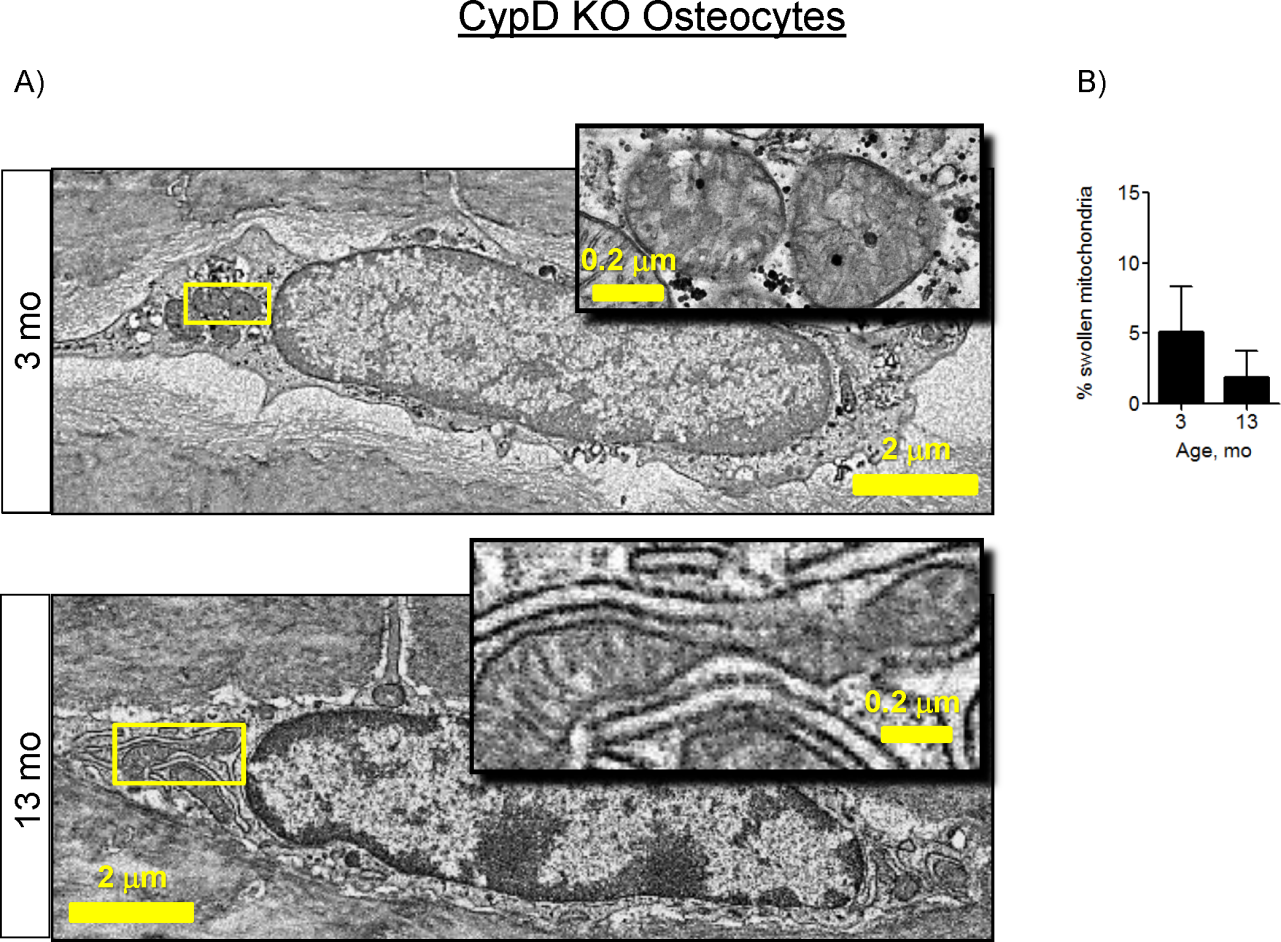
CypD deletion protects against osteocyte mitochondrial swelling in bone samples from 13-mo-old mice. A) Electron micrographs of osteocytes (OTs) in mouse tibia. Arrowheads indicate mitochondria. Boxed mitochondria are enlarged and shown in the insets; B) Quantitative analysis of mitochondrial morphology. Fifteen cells per sample and 3 mice per group were blindly analyzed by three independent scorers and the number of mitochondria in a swollen conformation was determined. Data are Means ± SD. *, *p*<0.05 as determined with *t*-test.

Since we observed a significant increase in the number of swollen mitochondria in 13-mo wild type C57BL/6J OTs (Fig 4), we examined the tibial bones of CypD KO mice with electron microscopy and detected no significant mitochondrial swelling in OTs (Fig 8). Altogether, these data indicate that, in contrast to the wild type C57BL/6J mice, there were no significant changes in bone cell energy metabolism and mitochondria in 13-mo-old CypD KO mice. Therefore, CypD deletion protects bone cell metabolism from age-associated changes, suggesting a causative role for the MPTP in these changes.

## Discussion

Here we show that bone loss in aging C57BL/6J mice coincides with metabolic changes in bone tissue indicative of mitochondrial dysfunction. We also observed increased mitochondrial swelling in osteocytes, indicating increased MPTP activity. Importantly, in mice with MPTP loss-of-function (CypD KO), we observed increased resistance against age-associated bone loss. CypD deletion also prevented the decline in bone formation, glycolytic shift and other metabolic changes in bone tissue indicative of impaired oxidative metabolism as well as mitochondrial swelling. There was also an effect of CypD deletion on bone resorbing OCs and a more pronounced decline in TRAP positive OCs in 13-mo old CypD KO mice than in 13-mo old wild type C57BL/6J mice. This effect requires further detailed investigation, however previous studies [29] already showed inverse correlation between mitochondrial activity and OC function. Thus, improved mitochondrial function due to CypD deletion could be a reason for accelerated OC decline in aging in CypD KO mice.

While cells have multiple ways to produce energy, the most efficient is via mitochondrial oxidative phosphorylation. Hence, BMSCs upregulate oxidative phosphorylation during differentiation into the osteogenic lineage [19–21]. Glycolytic metabolism was found to be equally important for the osteogenic lineage [30,31], however, these findings by no means imply that mitochondria are not critical to bone physiology. Glycolysis dominance over OxPhos may be required at the initial proliferative stages of osteogenic differentiation, while activation of mitochondria may be needed for massive protein biosynthesis at later stages. These different findings indicate the metabolic plasticity of bone cells and suggest that further detailed studies of metabolic mechanisms in bone cells are required.

There is still much to learn about the underlying mechanisms of bone loss during aging. Age-associated bone loss was previously thought to be primarily due to hormone depletion, such as the decline in estrogen production after menopause in females. More current opinions hold that this may not be the only cause, and that oxidative stress may also have strong impact [17,18]. Both of these elements are detrimental to bone, but their respective contributions have not been clarified. Importantly, both estrogen depletion and oxidative stress have harmful effects on mitochondria by inducing the MPTP [12,32–35]. Since this study was performed on male mice of moderate age, it is likely that oxidative stress was more relevant than hormone depletion. Pathological levels of oxidative stress (ROS) can activate CypD and, thus, promote the MPTP opening [16]. Oxidative stress found in aging bone tissue may damage mitochondria via the MPTP in bone cells, leading to decreased cell function and disruption of tissue homeostasis. The cause and effect relationship between ROS and MPTP in bone require detailed investigation and is a subject of our ongoing studies.

We assessed changes in aging bone using multiple techniques, such as microCT, histomorphometry, and biomechanical testing. These assays clearly show the age-associated decrease in bone and impaired bone formation in wild type C57BL/6J mice, and lack thereof in CypD KO mice. Currently, the most informative technique that allows a snapshot of energy metabolism in whole tissue is metabolomics. We therefore used this technique to assess the metabolic profile of bone and detected a glycolytic shift indicative of mitochondrial dysfunction in aging wild type C57BL/6J, but not in CypD KO mice. To our knowledge, this is first time this technique has been applied to bone tissue. OTs comprise more than 70% of the cellular component of the bone tissue that was used in our metabolomic assay, and therefore, it is reasonable to assume that the metabolic changes observed in 13-mo-old wild type C57BL/6J mice were due to changes in OTs. This is further indicated by detection of mitochondrial swelling by EM in wild type C57BL/6J OTs and not in OBs. As evident from previously published works from our group and others [19–21], mitochondria are activated late in the course of osteogenic differentiation. Therefore while more work is needed to elucidate the contribution of different cell types, it is likely that mitochondria in OTs are most active and, thus, most vulnerable to the MPTP opening.

We have not yet examined mice of advanced age, i.e. older than 24 mo, and thus cannot claim if MPTP inhibition via CypD KO can protect bone during advanced aging. Our data in 18-mo-old mice show that the protective effect of CypD deletion is somewhat diminished compared to 13-mo-old mice, and so it is possible that CypD deletion cannot completely protect bone in aging, and instead delay the onset of osteoporosis. Despite the fact that our CypD KO mice are of C57BL/6J background, our mice were not littermates and were bred in different facilities. This was a major limitation of our study that did not allow us to directly compare CypD KO mice to their wild type counterparts. Additionally, this work was done in global KO mice, so the observed effects could be caused by systemic changes induced by CypD deletion. However, a global KO is clinically relevant as a pharmacological CypD inhibitor would produce a similar systemic effect.

To our knowledge, this is the first study using metabolomics in bone tissue and showing that protecting mitochondrial function is linked to improved bone health in aging. This opens a broad and overlooked target for new bone therapeutics: mitochondrial metabolism.

## Supporting Information

S1 Fig C57BL/6J mice lose bone at 13- and 18-mo of age. Quantitative volumetric analysis of microCT data. Data are Means ± SD (n = 5–15). *, *p*<0.05 vs 3 mo as determined with ANOVA.
(TIFF)

S2 Fig C57BL/6J mice decrease cortical bone formation at 13-mo of age. Quantitative analyses of mouse bones labeled with Alizarin Red and Calcein for dynamic Mineral Apposition Rate (MAR), Mineralizing Surface/Bone Surface (MS/BS) and Bone Formation Rate (BFR) assay. Data are Means ± SD (n = 5). *, *p*<0.05 vs 3 mo as determined with *t-*test.
(TIFF)

S3 Fig Metabolic change in bone samples from 18-mo-old C57BL/6J mice. Small metabolites were extracted from bone shafts of tibia and femurs and analyzed using metabolomic LC-MS. Metabolites are grouped into appropriate metabolic pathways. Data are Means ± SD (n = 3). *, *p*<0.05 vs 3 mo as determined with *t-*test.
(TIFF)

S4 Fig No mitochondrial swelling in osteoblasts in bones from 13-mo-old C57BL/6J mice. Electron micrographs of osteoblasts in mouse tibia. Arrowheads indicate mitochondria.
(TIFF)

S5 Fig CypdKO mice do not lose bone at 13- and 18-mo of age. Quantitative volumetric analysis of microCT data. Data are Means ± SD (n = 5–15). *, *p*<0.05 vs 3 mo as determined with ANOVA.
(TIFF)

S6 Fig CypdKO mice do not decrease cortical bone formation at 13-mo of age. Quantitative analyses of mouse bones labeled with Alizarin Red and Calcein for dynamic Mineral Apposition Rate (MAR), Mineralizing Surface/Bone Surface (MS/BS) and Bone Formation Rate (BFR) assay. Data are Means ± SD (n = 5). *, *p*<0.05 vs 3 mo as determined with *t-*test.
(TIFF)

S7 Fig No metabolic change in bone samples from 18-mo-old CypDKO mice. Small metabolites were extracted from bone shafts of tibia and femurs and analyzed using metabolomic LC-MS. Metabolites are grouped into appropriate metabolic pathways. Data are Means ± SD (n = 3). *, *p*<0.05 vs 3 mo as determined with ANOVA.
(TIFF)

S1 Table Osteoblast parameters in 3- and 13-mo-old C57BL/6J wild type mice. (TIFF)

S2 Table Metabolite levels in 3-mo-old mice. (TIFF)
