# Supplementary figures and images for: Cyclophilin D Knock-Out Mice Show Enhanced Resistance to Osteoporosis and to Metabolic Changes Observed in Aging Bone
Source: PLoS One. 2016 May 16;11(5):e0155709. doi: 10.1371/journal.pone.0155709 (PMC4868300; doi:10.1371/journal.pone.0155709)

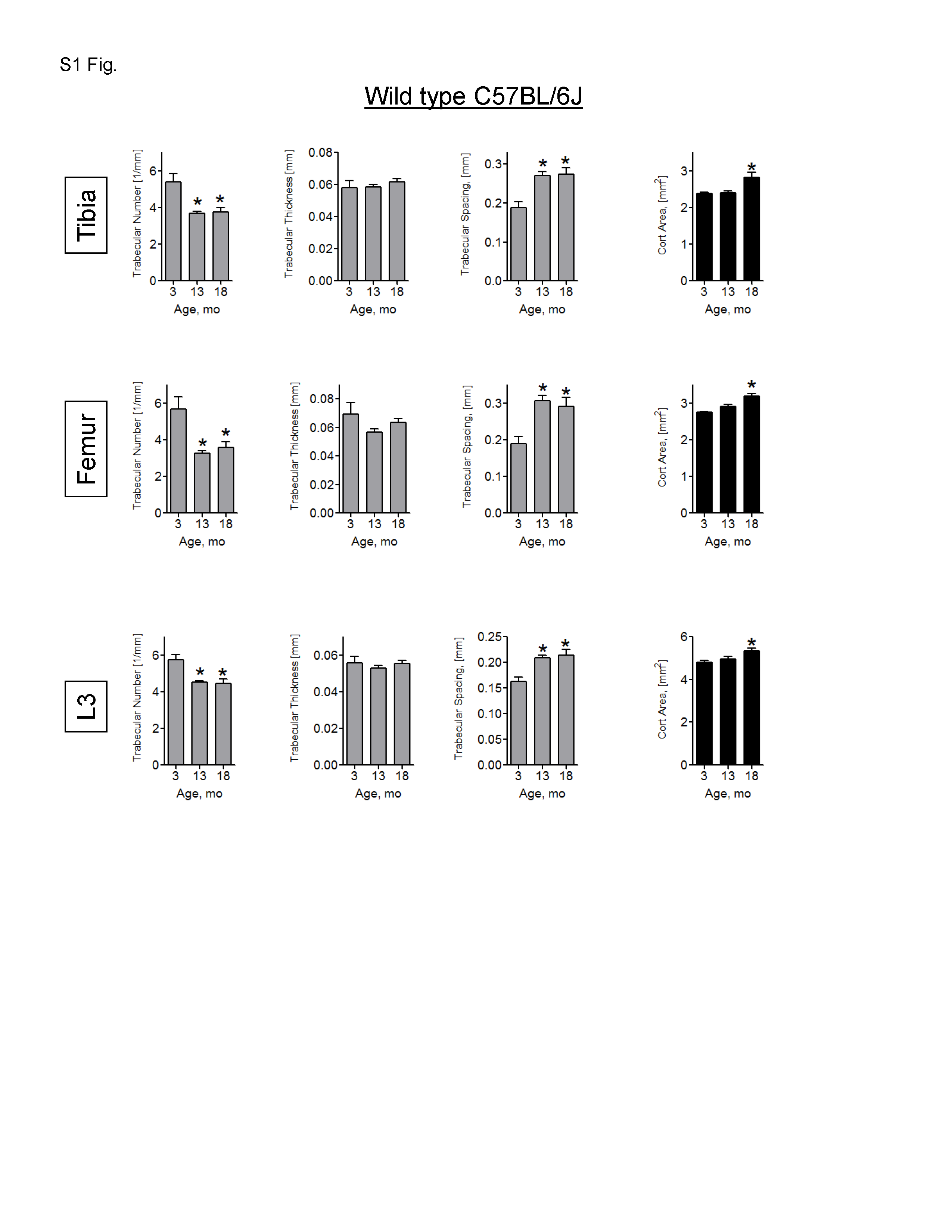

Supplement: S1 Fig — Quantitative volumetric analysis of microCT data. Data are Means ± SD (n = 5–15). *, p<0.05 vs 3 mo as determined with ANOVA. (TIFF) [file pone.0155709.s001.tiff]

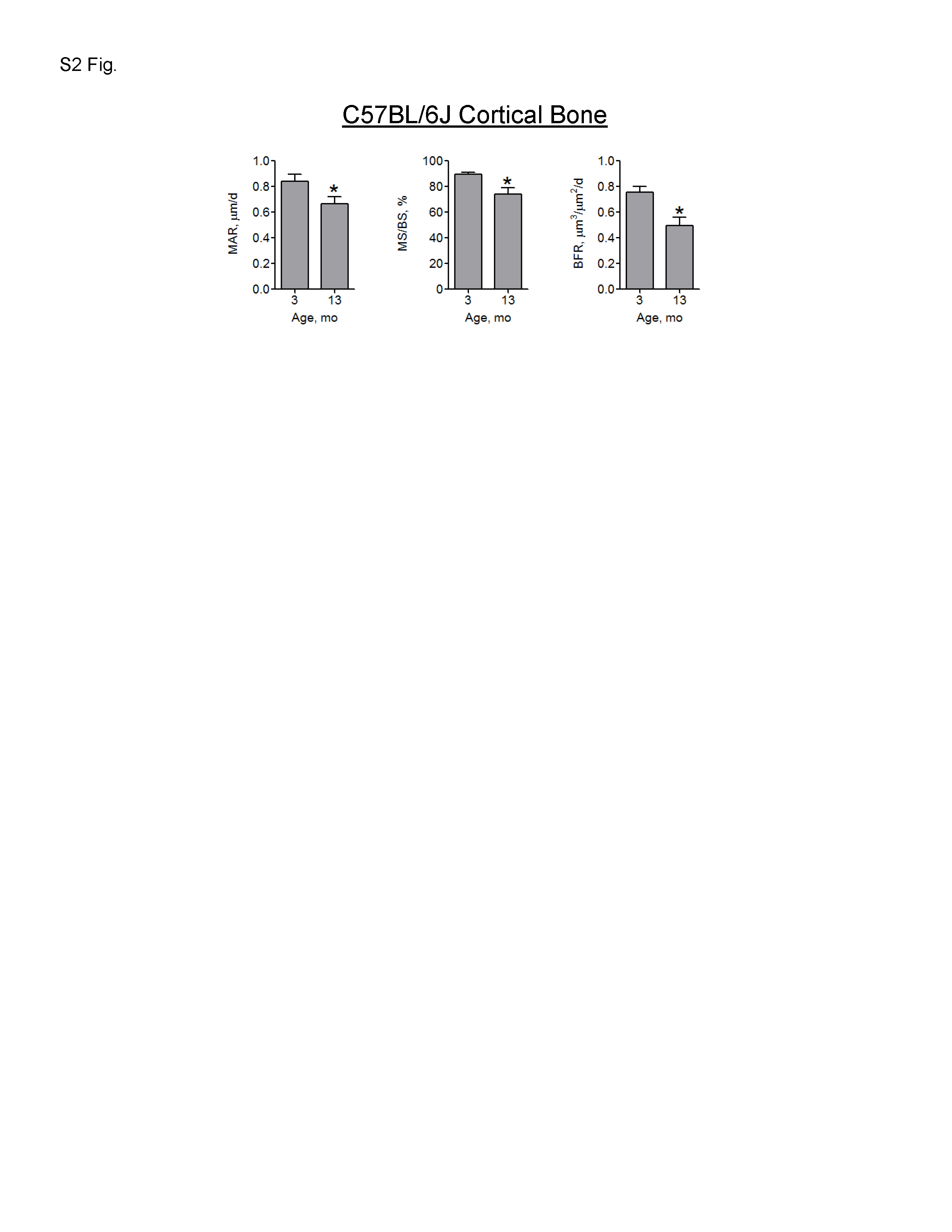

Supplement: S2 Fig — Quantitative analyses of mouse bones labeled with Alizarin Red and Calcein for dynamic Mineral Apposition Rate (MAR), Mineralizing Surface/Bone Surface (MS/BS) and Bone Formation Rate (BFR) assay. Data are Means ± SD (n = 5). *, p<0.05 vs 3 mo as determined with t-test. (TIFF) [file pone.0155709.s002.tiff]

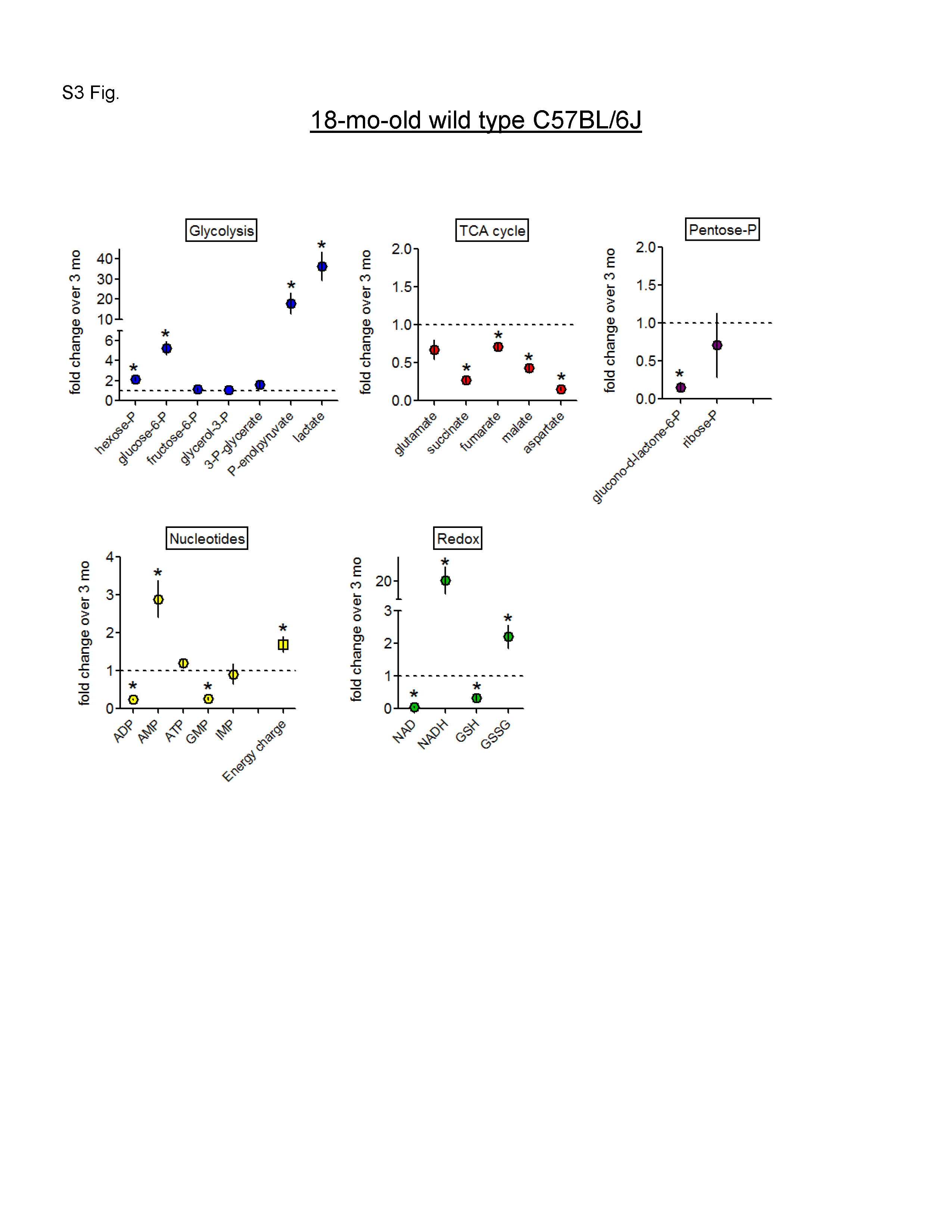

Supplement: S3 Fig — Small metabolites were extracted from bone shafts of tibia and femurs and analyzed using metabolomic LC-MS. Metabolites are grouped into appropriate metabolic pathways. Data are Means ± SD (n = 3). *, p<0.05 vs 3 mo as determined with t-test. (TIFF) [file pone.0155709.s003.tiff]

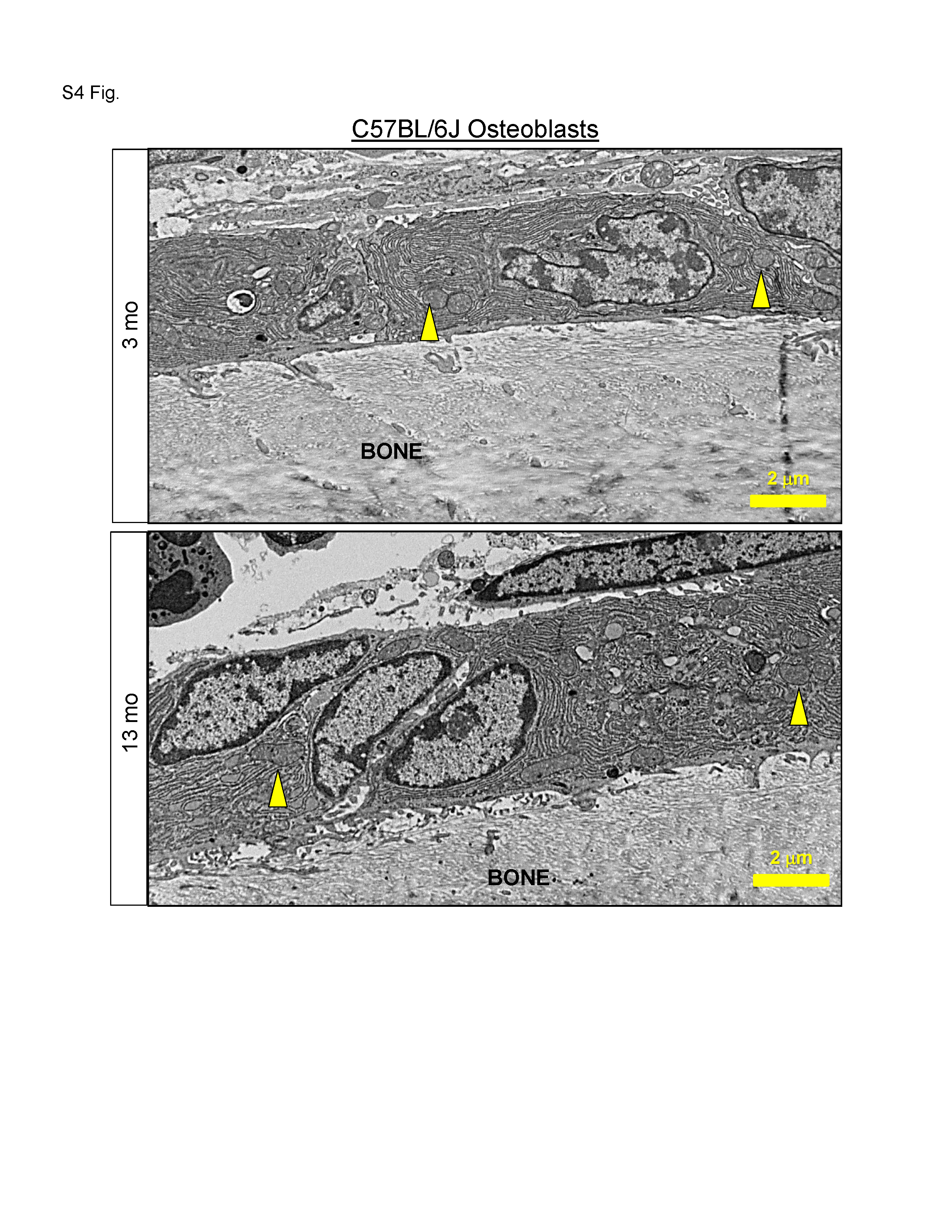

Supplement: S4 Fig — Electron micrographs of osteoblasts in mouse tibia. Arrowheads indicate mitochondria. (TIFF) [file pone.0155709.s004.tiff]

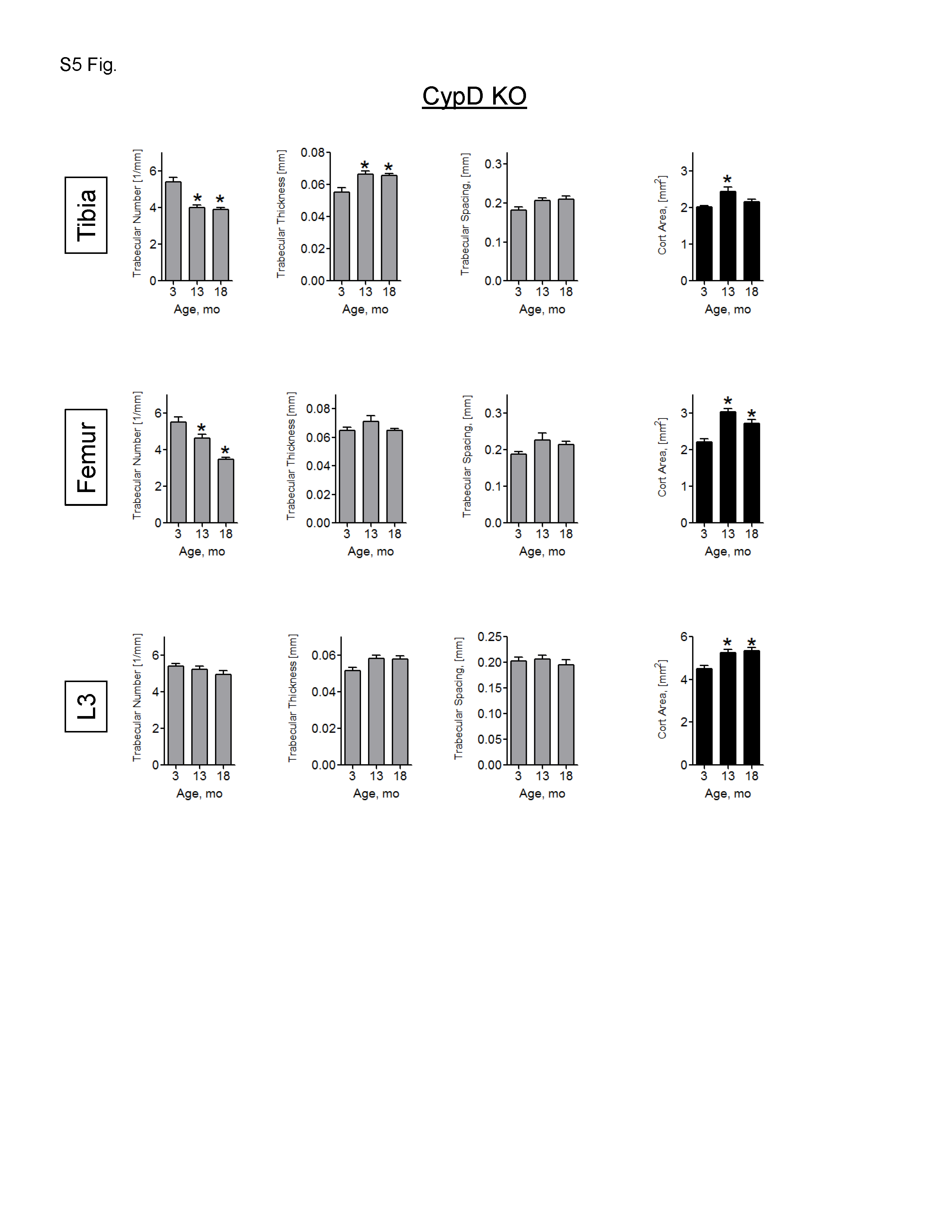

Supplement: S5 Fig — Quantitative volumetric analysis of microCT data. Data are Means ± SD (n = 5–15). *, p<0.05 vs 3 mo as determined with ANOVA. (TIFF) [file pone.0155709.s005.tiff]

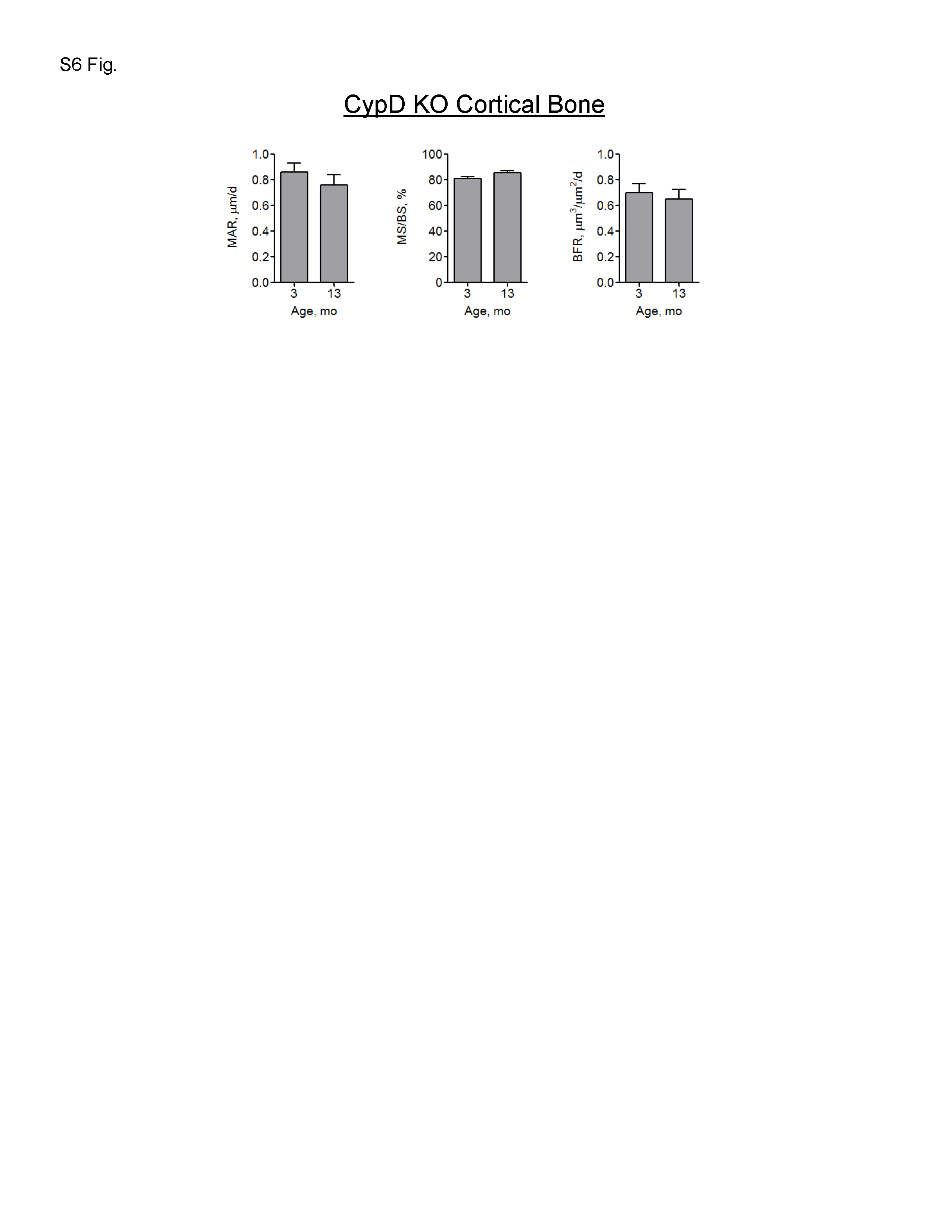

Supplement: S6 Fig — Quantitative analyses of mouse bones labeled with Alizarin Red and Calcein for dynamic Mineral Apposition Rate (MAR), Mineralizing Surface/Bone Surface (MS/BS) and Bone Formation Rate (BFR) assay. Data are Means ± SD (n = 5). *, p<0.05 vs 3 mo as determined with t-test. (TIFF) [file pone.0155709.s006.tiff]

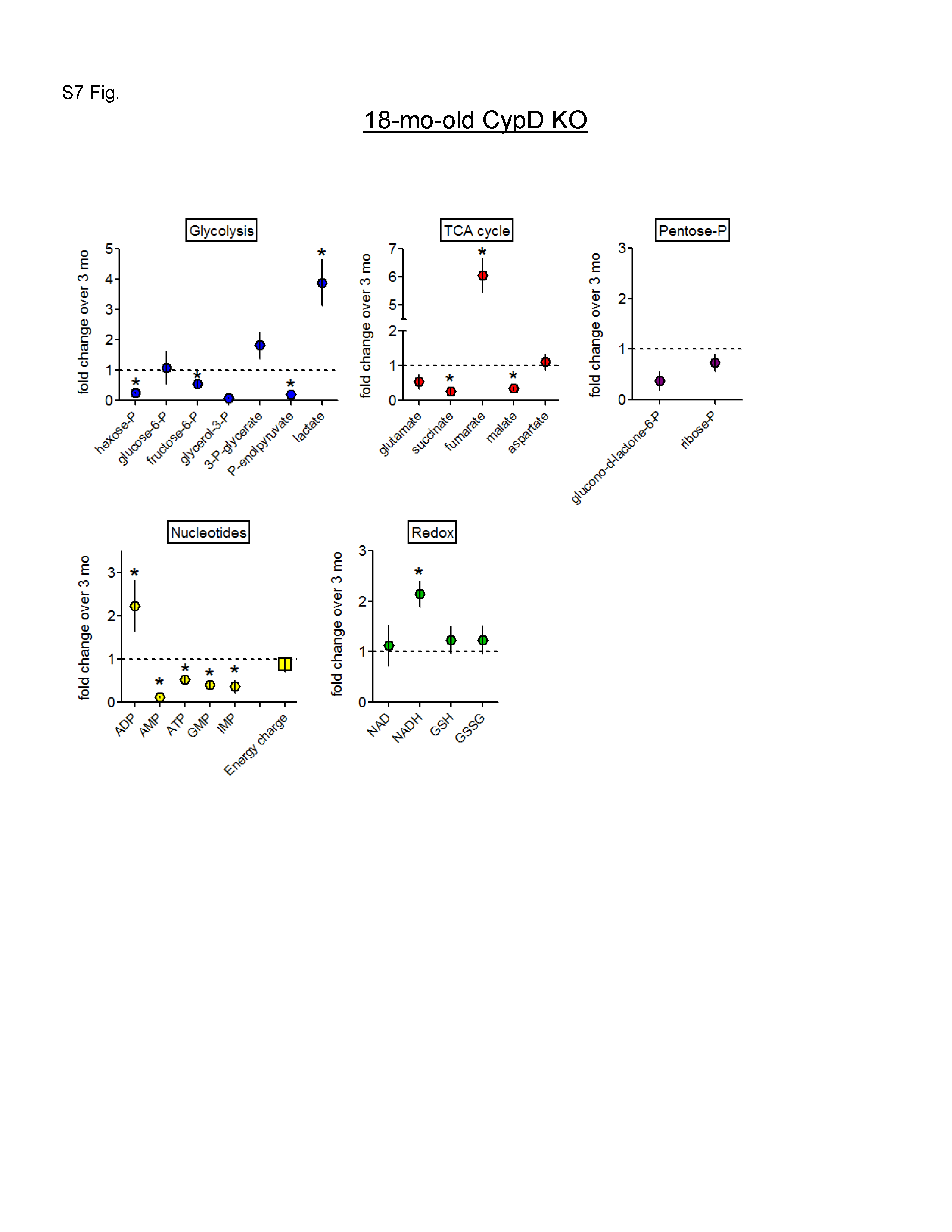

Supplement: S7 Fig — Small metabolites were extracted from bone shafts of tibia and femurs and analyzed using metabolomic LC-MS. Metabolites are grouped into appropriate metabolic pathways. Data are Means ± SD (n = 3). *, p<0.05 vs 3 mo as determined with ANOVA. (TIFF) [file pone.0155709.s007.tiff]

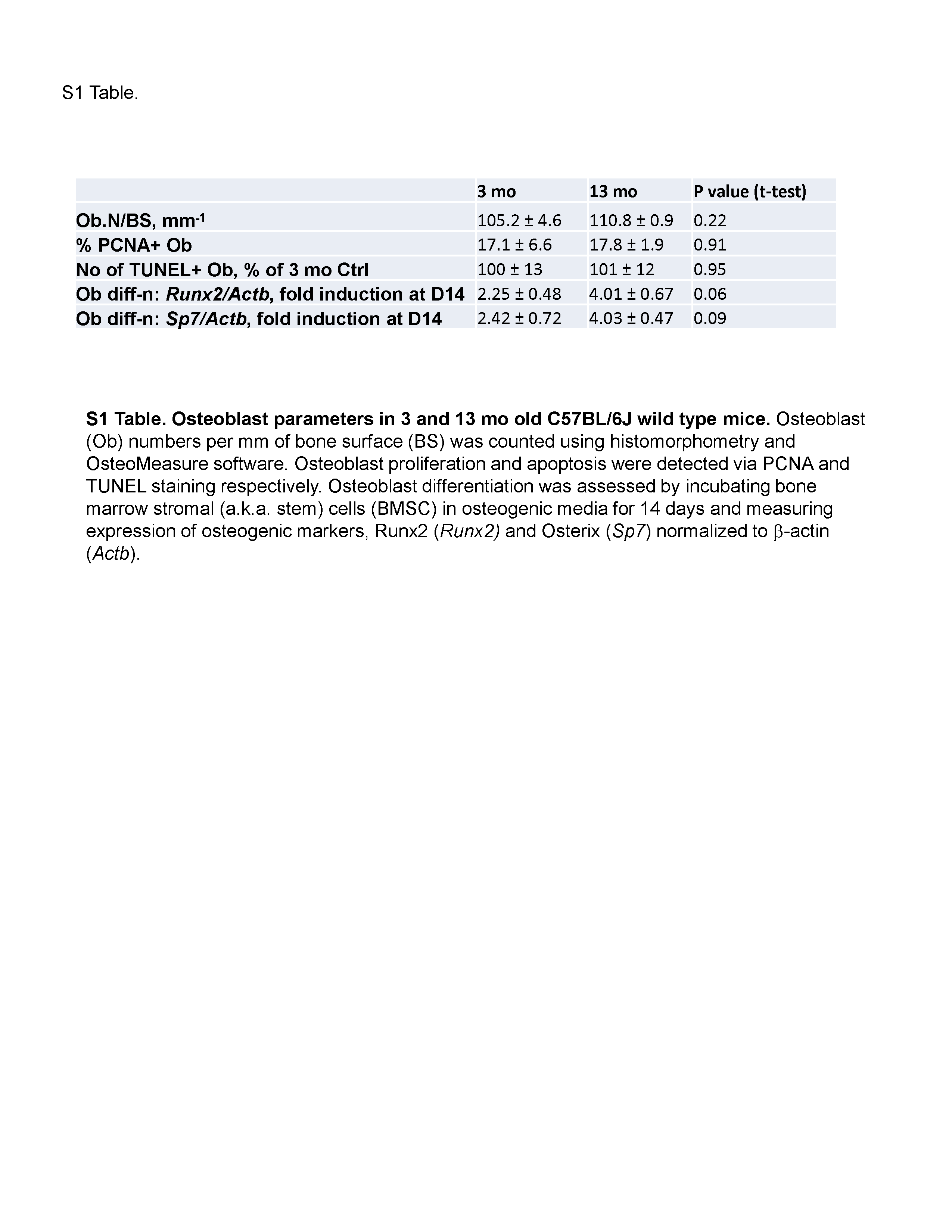

Supplement: S1 Table — (TIFF) [file pone.0155709.s008.tiff]

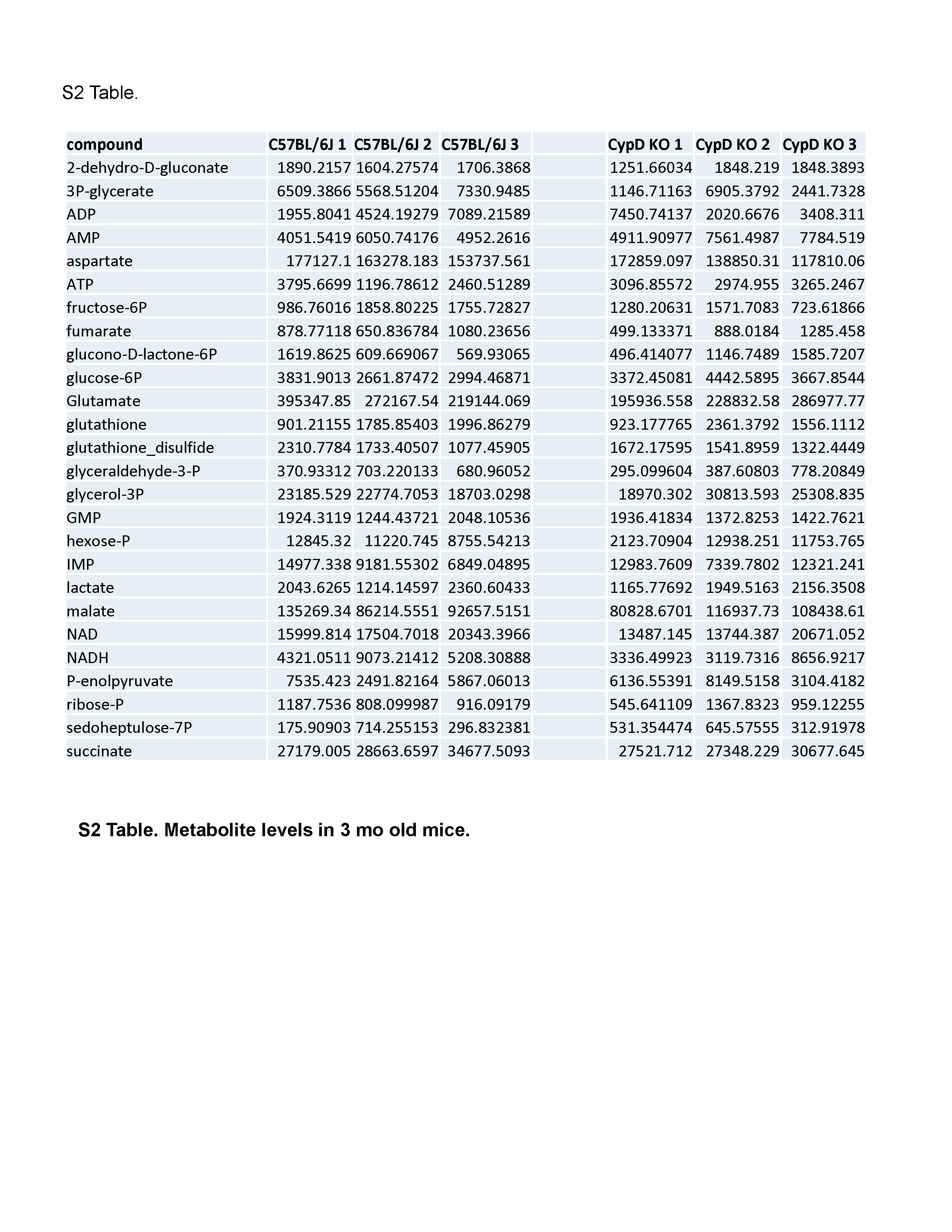

Supplement: S2 Table — (TIFF) [file pone.0155709.s009.tiff]
